# Behçet’s Disease: A Comprehensive Review on the Role of HLA-B*51, Antigen Presentation, and Inflammatory Cascade

**DOI:** 10.3390/ijms242216382

**Published:** 2023-11-16

**Authors:** Saba Khoshbakht, Defne Başkurt, Atay Vural, Seçil Vural

**Affiliations:** 1 Koç University Research Center for Translational Medicine, Istanbul 34010, Turkey; skhoshbakht20@ku.edu.tr (S.K.); atayvural@ku.edu.tr (A.V.); 2 School of Medicine, Koç University, Istanbul 34010, Turkey; dbaskurt17@ku.edu.tr; 3 Department of Neurology, Koç University School of Medicine, Istanbul 34010, Turkey; 4 Department of Dermatology and Venereology, Koç University School of Medicine, Istanbul 34010, Turkey

**Keywords:** Behçet’s disease, HLA-B*51, ERAP, pathogenesis, T cell receptor, antigens

## Abstract

Behçet’s disease (BD) is a complex, recurring inflammatory disorder with autoinflammatory and autoimmune components. This comprehensive review aims to explore BD’s pathogenesis, focusing on established genetic factors. Studies reveal that HLA-B*51 is the primary genetic risk factor, but non-HLA genes (*ERAP1*, *IL-10*, *IL23R/IL-12RB2*), as well as innate immunity genes (*FUT2*, *MICA*, *TLRs*), also contribute. Genome-wide studies emphasize the significance of *ERAP1* and HLA-I epistasis. These variants influence antigen presentation, enzymatic activity, and HLA-I peptidomes, potentially leading to distinct autoimmune responses. We conducted a systematic review of the literature to identify studies exploring the association between HLA-B*51 and BD and further highlighted the roles of innate and adaptive immunity in BD. Dysregulations in Th1/Th2 and Th17/Th1 ratios, heightened clonal cytotoxic (CD8+) T cells, and reduced T regulatory cells characterize BD’s complex immune responses. Various immune cell types (neutrophils, γδ T cells, natural killer cells) further contribute by releasing cytokines (IL-17, IL-8, GM-CSF) that enhance neutrophil activation and mediate interactions between innate and adaptive immunity. In summary, this review advances our understanding of BD pathogenesis while acknowledging the research limitations. Further exploration of genetic interactions, immune dysregulation, and immune cell roles is crucial. Future studies may unveil novel diagnostic and therapeutic strategies, offering improved management for this complex disease.

## 1. Introduction

Behcet’s disease (BD) is a recurrent and multisystem inflammatory disease that was first described by Dr. Hulusi Behçet, a Turkish dermatologist, in 1937 [1]. The disease typically affects individuals in their third to fourth decade of life and does not display a gender preference. However, severe morbidities are more prevalent in men [2]. With age, the disease activity tends to decrease after severe repetitive attacks in the young productive population. Therefore, BD imposes a considerable economic burden on society. BD is considered an orphan disease in the Western world due to its low prevalence (1–10/100,000), whereas in many developing countries, such as Turkey, BD is more common, with a prevalence of 0.4–4/1000. Japan and Middle Eastern countries also have a higher prevalence of BD [3,4]. 

Currently, BD is diagnosed only based on clinical findings and there is no definitive laboratory test to confirm the diagnosis. According to The International Study Group criteria published in 1990 for BD, oral lesions plus two of uveitis, genital lesions, other skin lesions, and a positive skin pathergy test (SPT) are required for BD diagnosis [4]. Although mucocutaneous symptoms are the most common presenting symptoms [5], patients may exhibit vasculitis and multisystemic involvement resulting in significant morbidity [3,6]. Vascular involvement is a defining feature of BD, and both venous and arterial involvement are unique features of the disease. Patients may experience recurrent deep vein thromboses, arterial aneurysms, and intracardiac thromboses [6]. Colchicine is the first-line therapy for mucocutaneous symptoms, and immunosuppressive or immunomodulatory treatments are administered in refractory cases or patients with systemic disease involvement [3,6]. However, current therapeutic techniques may be insufficient in some individuals to control recurring episodes. 

BD is a mixed-pattern disease characterized by both autoinflammatory and autoimmune features that develop in genetically susceptible individuals exposed to environmental factors [7]. The genetic background is strong in BD pathogenesis. Familial aggregation is substantial with risk ratios ranging from 11.4 to 52.5 among siblings in different populations within Turkey [8,9]. A previous study in Korea, with over 21 million individuals in 12 million families, found that the risk of developing Behçet’s disease (BD) was significantly higher among first-degree relatives, especially for twins, with a 165-fold increase [10]. 

The most significant genetic association related to disease risk is the presence of the human leukocyte antigen (HLA) class I allele HLA-B*51. In recent years, genetic variations in non-HLA genes such as the endoplasmic reticulum aminopeptidase enzyme (ERAP1), interleukin-10 (IL-10), and interleukin-23 receptor/interleukin-12 receptor beta-2 (IL23R/IL-12RB2) have been identified as susceptibility loci for BD [4]. In individuals with genetic susceptibility, external factors such as microbial agents or internal self-antigens like heat shock proteins are suggested as triggers of autoimmune responses, leading to systemic inflammation and the clinical manifestations of BD [3,11]. Immune response aberrations result in the activation of a cytokine cascade and alterations in the levels of cytokines, driving inflammatory cells to migrate to the tissues, causing damage [11]. 

The current literature points out the role of both innate first-responder cells such as neutrophils and adaptive immune cells such as lymphocytes. The goal of this review is to give a comprehensive assessment of the studies on BD and HLA class I association and discuss the pathophysiology of BD, with an emphasis on HLA, genetics, and inflammation. 

## 2. Methods

We performed systematic literature research on PubMed and Google Scholar, searching for eligible records up to October 30, 2023. We searched the electronic databases for relevant articles with the keywords “*HLA*” or “HLA-B*51” or “MHC class I” or “GWAS” AND “Behcet” or “Behçet” or “Behçet’s”. EndNote 20 was used to collect the references. 

**Figure 1:**
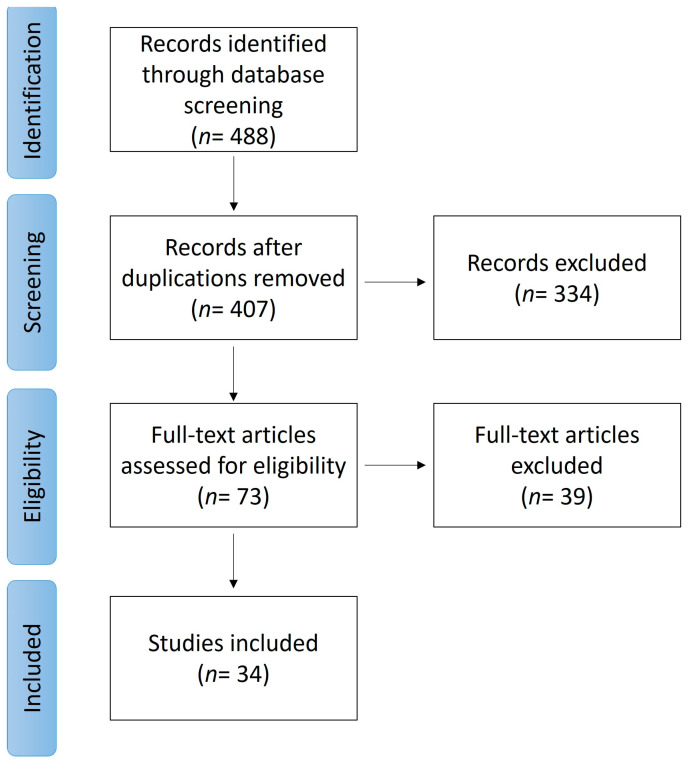
Flowchart of study selection according to PRISMA guidelines.

The initial search yielded 488 references excluding review papers. After removing duplicates, 407 citations remained. Following title and abstract screening, 334 reports were considered irrelevant. Consequently, 73 articles underwent a complete review. We finally included case-control studies, retrospective studies, meta-analyses, and genome wide association studies investigating HLA-B*51 association with BD. The full-text review resulted in the exclusion of 39 studies, and 34 articles remained for the final analysis. Figure 1 shows the study selection process.

## 3. Results

### 3.1. HLA-B*51:01 Association: The Strongest Genetic Risk Factor

The role of genetic factors in BD has been widely studied, and the HLA-B*51 allele has been identified as the primary risk factor for the disease [12,13]. Furthermore, the presence of this gene in populations around the globe correlates with the prevalence of BD. Our search revealed 34 studies, which are summarized in Table 1.

In Silk Road nations, up to 20–25% of the general population and 50–80% of BD patients carry the HLA-B*51 allele. In contrast, in the USA and northern Europe, HLA-B*51-positive individuals are 2–8% of the general population and about 15% of BD patients [14,15]. In Turkey, the prevalence of the HLA-B*51 allele in BD patients is high, ranging from 50% to 70%, whereas in the general population, around 25% of individuals are HLA-B*51-positive [16,17,18,19,20,21]. Carrying the HLA-B*51 allele increases the risk of developing BD almost 6–10-fold, with a risk ratio of 5.90 for HLA-B*51 carriers [19,20,22]. The clinical subtypes associated with HLA-B*51 carriage status have been investigated. Although the findings differ, presumably due to the different ethnicities in studies, a higher prevalence of ocular disease, vasculitis, neurological involvement, and a more severe phenotype are reported [17,23,24,25,26,27,28,29]. Kirino et al. [30] have reported that BD has evolved in recent years and both complete-type BD and the prevalence of HLA-B*51 positivity have decreased.

The presence of the HLA-B*51 allele is thought to influence the BD peptidome, leading to the presentation of specific self-antigens or microbial antigens that may trigger an autoimmune response [11]. However, the precise mechanisms by which HLA-B*51 contributes to the pathogenesis of BD are not yet fully understood.

**Table 1:** Studies investigating the association between HLA-B*51 and Behçet’s disease.

Reference	Study	Ethnicity	Patients	Results
Kim et al., 2018 [31]	R	Korean	OA: 433 BD: 126	In HLA-B*51+ BD patients (*n* = 40), clinical features of the diagnostic criteria were dominant In HLA-B*27+ BD patients (*n* = 17), genital ulcers and skin lesions were dominant
Krause et al., 1999 [29]	R	Israeli	BD: 55	*HLA-B5*+ patients have enhanced occurrence of thrombophlebitis, less erythema nodosum, older disease onset age, and more severe disease
Mizuki et al., 2020 [28]	R	Japanese	BD: 3044	HLA-B*51+ BD patients have: Increased risk of ocular lesion (OR 1.59, 95% CI: 1.37–1.84; *p* < 0.001) Decreased risk of genital ulceration (OR 0.72, 95% CI: 0.62–0.84; *p* < 0.001) and gastrointestinal symptoms (OR 0.65, 95% CI: 0.55–0.77; *p* < 0.001)
Pamukcu et al., 2022 [27]	R	Turkish	BD: 204	HLA-B*51+ BD patients have a higher risk of PPL (OR 1.946, 95% CI: 1.044–3.629) and ocular (OR 2.399, 95% CI: 1.165–4.938) and neurological involvement (OR 5.404, 95% CI: 1.119–26.093)
Rajaei et al., 2020 [18]	R	Iranian	BD: 63	The percentage of *HLA-B5* (25%) and HLA-B*51 (21%)
Ryu et al., 2018 [26]	R	Korean	BD: 193	HLA-B*51+ patients: Earlier disease onset (28.3 ± 11.4 years vs. 33.8 ± 11.6 years, *p* = 0.02), More frequent neurologic (17.2% vs. 2.5%, *p* = 0.02) and gastrointestinal involvements (20.7% vs. 2.5%, *p* = 0.01)
Ideguchi et al., 2011 [17]	R	Japanese	BD: 412	HLA-B*51 was positive in 50% (53% in male patients, 48% in female patients) Higher frequency of HLA-B*51 in patients with ocular involvement
Kirino et al., 2016 [30]	R	Japanese	BD: 578	Phenotypical evolution in Japanese BD patients during the last 30 years: Significant decrease in complete-type BD, HLA-B*51 carriers, and gastrointestinal symptoms
Soejima et al., 2021 [32]	R	Japanese	BD: 657 (1990–2018) BD: 6754 (2003–2014)	Temporary alteration of clinical cluster proportions over time caused increasing GI involvement, reduced incidence of complete type according to Japanese criteria, and reduced HLA-B*51-positive BD patients
Ortiz-Fernández et al., 2016 [33]	CC	Spanish	BD: 278 HC: 1517	Highest association with BD: HLA-B*51 (*p* = 6.82 × 10^-32^, OR 3.82) HLA-B57 (*p* = 1.02 × 10^-5^, OR 2.80, 95% CI = 1.77–4.43) and HLA-A03 (*p* = 9.68 × 10^-3^, OR 0.61, 95% CI = 0.41–0.89) identified as additional HLA genes associated with BD
Al-Okaily et al., 2016 [34]	CC	Saudi	BD: 60 HC: 60	Enhanced frequency of HLA-A*26, -A*31, and -B*51 alleles in BD patients HLA-B*15 allele may have a protective effect on BD
Alpsoy et al., 1998 [16]	CC	Turkish	BD: 71 HC: 600	HLA-B*51 significantly increased in BD patients *DR7* significantly decreased in BD patients
Demirseren et al., 2014 [35]	CC	Turkish	BD: 51 HC: 44	HLA-B*51 is significantly higher in BD patients HLA-B*5101, HLA-B*5102(01), HLA-B*5109, and HLA-B*5122 subtypes increased in BD patients Negative correlation between PPL involvement and HLA-B*5109 HLA-B*5103 may be a risk factor for neuro-Behçet
Hamzaoui et al., 2012 [36]	CC	Tunisian	BD: 178 HC: 125	Higher HLAB-51 frequency in BD patients (47.19% vs. 20.8%, *p* < 0.001). HLA B51+ patients have: Higher frequency of pathergy test positivity (*p* = 0.01) Retinal vasculitis (*p* = 0.045) Lower frequency of arterial aneurysms (*p* = 0.009) and neurological involvement
Itoh et al., 2006 [21]	CC	Japanese	BD: 180 HC: 170	Strong association of HLA-B*5101 with BD (Pc = 1 × 1016, OR = 8.5) Weak association between A2602 (Pc = 0.130, OR = 4.3) and HLA-B3901 (Pc = 0.099, OR = 3.5)
Koumantaki et al., 1998 [20]	CC	Greek	BD: 62 HC: 87	Higher frequency of HLA-B*5101 in BD patients (80% vs. 26%) (OR 10.48, *p* < 10^−6^) Males carrying B5101 allele have a higher risk for BD compared to females (OR 16.97 vs. 5.74, respectively)
Mizuki et al., 2001 [37]	CC	Jordanian	BD: 49 HC: 50	The strongest risk factor: HLA-B*51
Castillo-Palma, et al., 1996 [38]	CC	Spanish	BD: 67 HC: 223	HLA-B*51+ is higher in males with ocular (*p* = 0.0001), cutaneous (*p* = 0.001), and digestive involvement (*p* = 0.05) DQB1*0303 was linked to worse prognosis in uveitis (*p* = 0.01) DR11 and DQB1*0301 were more common in HLA B51+ patients
Muñoz et al., 2020 [39]	CC	Argentinian	BD: 34 HC: 240	BD was associated with HLA-B*51 allele (OR = 3.75; *p* = 0.0012).
Pirim et al., 2004 [22]	CC	Turkish	BD: 75 HC: 54	HLA-B*51 frequency is higher in BD (58.7% vs. 18.5%, OR = 6.245) Most prevalent class II HLA in BD were HLA-DRB1*04 (45.3%) and HLA-DRB1*07 (24%)
Rodríguez et al., 1998 [40]	CC	Spanish	BD: 21 HC: 25	HLA-B*51 frequency is higher in BD (37.5% vs. 15.5%) BD patients had higher frequencies of B*5101 (32% vs. 13%), and 5108 (5.5% vs. 1.2%)
Sakly et al., 2009 [41]	CC	Tunisian	AS&BD: 365 HC: 124	No significant difference in HLA-B*51 between BD and HC (30.0% vs. 16.1%, *p* > 0.05)
Sanz et al., 1998 [42]	CC	Spanish	BD: 56 HC: 66	Association of Cw*1602 with BD (OR 20.15, corrected ρ < 0.05) with higher relative risk compared to association of B51 in this study (OR 1.85)
Zouboulis et al., 1993 [25]	CC	German	BD: 39 HC: 1415	Higher frequency of HLA-B5 allele among BD patients (*p* < 0.05) Higher frequency of HLA-B5 in male patients with severe vascular involvement
Ombrello et al., 2014 [43]	CC	Turkish	BD: 1190 HC: 1257	HLA-B*51, -A*03, -B*15, -B*27, -B*49, -B*57, and -A*26 with risk of BD Independent associations between BD and the *HLA-B/MICA* region and the area between HLA-F and HLA-A (*p* < 1.7 × 10^−8^)
Capittini et al., 2021 [44]	Ma	Worldwide	NA	The most frequent HLA-B*51 two-digit alleles associated with BD differed among populations: Europe, HLA-B*5108 (OR 11.25 C.I. 4.9–26) Turkey, HLA-B*5101 (OR 5.98 C.I. 3.7–9.8) Japan, HLA-B*5102 (OR 5.39 C.I. 0.6–47) HLA class I alleles associated with risk for BD are B*5108, B*51, B*5101, B*5102, DQB1-03, A*2601, Cw14, Cw15, Cw16, B*15, and A*26
de Menthon et al., 2009 [19]	Ma	Worldwide	BD:4800 HC: 16289	Enhanced risk of BD 5.78 and 5.9 times over in carriers of HLA–B*51/B*5 and HLA–B*51, respectively
Maldini et al., 2012 [24]	Ma	Worldwide	NA	HLA-B*51/B5+ positivity is associated with: Male gender Increased frequencies of genital ulcers and ophthalmic and skin manifestations Reduced frequency of gastrointestinal involvement
Horie et al., 2017 [23]	Ma	Worldwide	NA	HLA-B*51 strongly associated with BD ocular manifestations in East and Middle Eurasian regions (OR = 2.40, *p* = 0.0030 and 1.87, *p* = 0.0045, respectively), but not in West Eurasian regions (*p* = 0.35)
Kirino et al., 2013 [45]	GWAS	Turkish	BD: 1209 HC: 1278	*ERAP1* variants preferentially conferred risk for BD in HLA-B*51-positive individuals (*p*-value = 0.0009) *ERAP1* p.Arg725Gln homozygosity was related with BD with an odds ratio of 3.78 [95% CI 1.94–7.35] in HLA-B*51-positive people and 1.48 [95% CI 0.78–2.80] in negative individuals New susceptibility loci detected at *CCR1, STAT4*, and *KLRC4* for BD
Remmers et al., 2010 [13]	GWAS	Turkish	BD: 1214 HC: 1278	Association of BD and HLA-B*51 (OR = 3.49, 95% CI = 2.95 to 4.12, *p* = 5.47 × 10^−50^) Identification of a second, independent association within the MHC class I region telomeric to HLA-B Association at IL10 (rs1518111, *p* = 1.88 × 10^−8^)
Su et al., 2022 [46]	GWAS	Chinese	BD: 1015 HC: 4502	Association of HLA–B*51, HLA–A*26, and HLA–C*0704 with BD-related uveitis 22 new susceptibility variations in 16 non-HLA loci (*RHOH, PRDM1, MTHFD1L, KLF4, ZMIZ1, RPS6KA4-PRDX5, SIPA1-FIBP-FOSL1, IL10RA, VAMP1, AGBL1, CMIP, CDH15-ZNF778, TCF4, MRPL39-JAM2, GART*, and *MIS18A*)

Abbreviations: R: Retrospective, CC: Case control studies, Ma: Meta-analysis, GWAS: Genome-wide association studies, BD: Behçet’s disease, HC: Healthy controls, OA: Oral aphthous lesions, AS: Ankylosing spondylitis, PPL: Papulopustular lesions.

### 3.2. HLA Class I Association in Antigen Presentation, TCR Antigen Recognition and Inflammation

Today, more than 19,000 HLA class I alleles have been identified, but only 4 of them were shown to have a substantial link with the risk of certain diseases known as major histocompatibility complex (MHC)-class I-opathy or MHC-I-related diseases. Among thousands of HLA class I alleles identified, only a few are linked to certain diseases, including HLA-B*51 for BD, HLA-B27 for the spondylarthritis family (ankylosing spondylitis (AS), psoriatic arthritis, reactive arthritis, arthritis associated with inflammatory bowel disease (IBD)), HLA-C06:02 for psoriasis, and HLA-A29:02 for birdshot chorioretinopathy. This highlights the significance of the HLA system in immune response and disease susceptibility [47,48,49,50]. 

**Figure 2:**
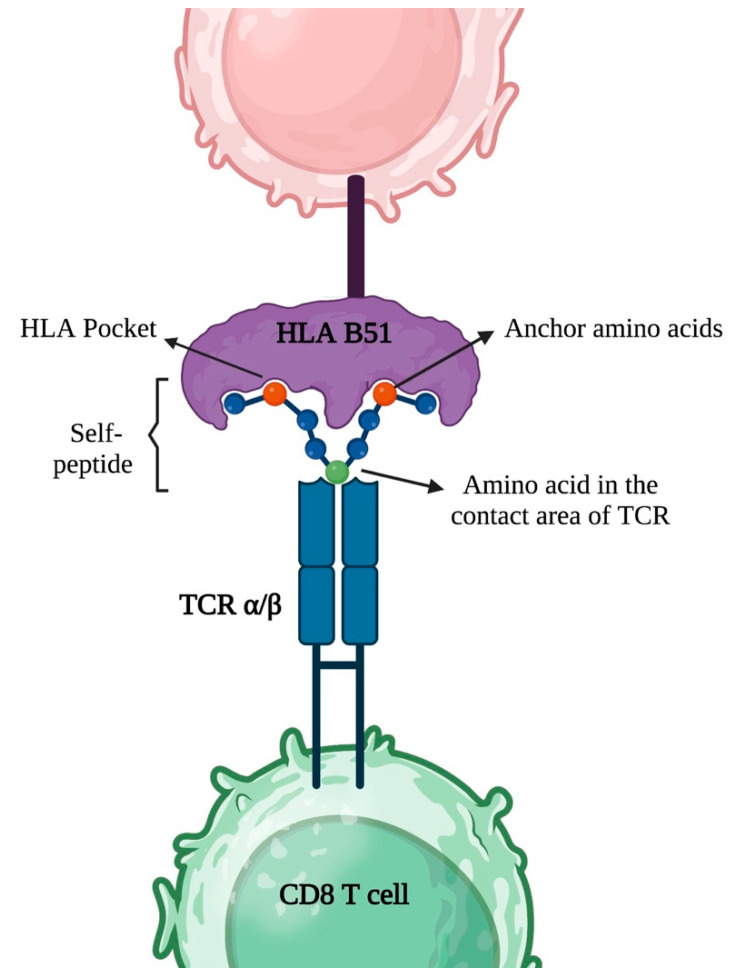
Schematic illustrates the process of HLA class I presentation of peptides containing 8–10 amino acids to the T cell receptor of CD8+ T cells. Anchor amino acids play a definitive role in peptide binding to HLA class I molecules. While T cell receptors exhibit polyspecificity, the specific contact residues on the peptide are of utmost importance in dictating the recognition. (Created using BioRender.com, accessed on 8 June 2023).

This peptide repertoire is determined by several factors, including the HLA molecule itself, the peptide-binding groove, the peptide-processing pathway, and the antigen-processing machinery as illustrated in Figure 2 [51]. The peptides presented by HLA molecules can originate from various sources, including self-, foreign, and viral antigens. Therefore, HLA molecules play a vital role in immune surveillance, immune activation, and immune regulation by presenting peptides to T cells for recognition and activation or tolerance induction [49]. 

The anchor residues within HLA class I molecules exhibit a high degree of rigidity and stability, playing a crucial role in peptide presentation. The peptides that are loaded onto and displayed by these molecules typically consist of 8 to 10 amino acids (aas) in length. The precise aa sequence pattern of these peptides is dictated by the distinctive configuration of HLA anchor residues. Although T cell receptors (TCRs) exhibit a poly-specific reactivity toward the antigens presented by HLA molecules, they can selectively recognize cognate HLA–peptide complexes via specific aa sequences. These sequences are determined jointly by the HLA anchor residues and the contact residues on the TCRs. Notably, the other positions within the peptide sequence offer flexibility for variation [52]. The genetic polymorphisms of HLA alleles contribute to the diverse array of peptide repertoires, leading to the generation of distinct peptidomes [44]. 

### 3.3. Gene Loci Related to Antigen Presentation 

#### 3.3.1. HLA-B*51 and ERAP1 Epistasis in Behçet’s Disease

Genome-wide association (GWA) studies have highlighted ERAP1, along with HLA-I, as a key genetic factor in BD [45,53]. In BD, an epistatic connection between HLA-B*51 and ERAP1 exists, making specific ERAP1 variations a risk factor only in the presence of the HLA-B*51 allele. These ERAP1 polymorphisms can impact antigen presentation and enzymatic activity, thus altering the HLA-I peptidome and binding affinity [11,12,24]. The ERAP1 and ERAP2 enzymes, operating in the endoplasmic reticulum, trim protease-degraded peptides for optimal HLA class I presentation, determining antigenicity [48,54]. ERAP1/2 polymorphisms are strongly associated with HLA-I-related conditions like BD, psoriasis, AS, and birdshot chorioretinopathy [54]. Notably, 10 primary ERAP1 variations (Hap1-10) exist, with Hap10 significantly linked to BD risk since its reduced activity leads to longer peptides [55,56,57]. A recent study by Cavers et al. [57] demonstrated that the ERAP1-Hap 10 allotype together with HLA-B*51 shifts the CD8+T cells from a naïve to an antigenic form, possibly displaying CD8+ T cell migration to inflamed tissues. 

The ERAP1-Hap10 variant, linked to higher BD risk, is strongly associated with HLA-B*51. Hap10 homozygotes with HLA-B*51 have a 10.96-fold BD susceptibility, while those lacking HLA-B*51 show no risk change [56]. Hap10’s reduced enzymatic activity leads to longer peptide trimming, especially noticeable with HLA-B*51-bound proteins [57]. Guasp et al. [58] compared the Hap10 alterations in HLA-B*51’s Ala-2 and Pro-2 subpeptidomes, resulting in a higher Ala-2/Pro-2 ratio, creating low-affinity HLA-B*51 peptides. This affects natural killer (NK) and cytotoxic T cell activity [48,58]. Low-affinity peptides with HLA-I can provoke cytotoxic T cell responses, while reduced activity of ERAP1 makes cells susceptible to NK cell killing due to a poor recognition of peptide–MHC-I complexes by KIRs [58,59].

The role of ERAP1 in the immune response of BD was investigated in HSV-induced BD mice with partial ERAP1 expression (ERAP1 heterozygotes, +/−). Lower ERAP1 expression enhanced the production of IL-17 and IFN-γ by the CD4+ T cells in BD mice [60]. A separate study also reported that ERAP1−/− mice exhibited decreased peripheral T regulatory cells which prevent autoreactive immune responses to self-antigens [61]. Thus, lower activity of ERAP1 impairs the Th1/Th17 and Treg cell balance in BD. These findings emphasize the intricate interaction between HLA-B*51 and ERAP1 and their significant impact on the process of antigen presentation, shedding light on the genetic factors contributing to the pathogenesis of BD. 

#### 3.3.2. The Repertoire of HLA-B*51 and Binding of Peptides

In a study by Gebreselassie et al. [62] the importance of peptidome and its association with BD was investigated. The study focused on the peptides associated with six different HLA-I alleles: *HLA-03*, HLA-A*31, HLA-B*18, HLA-B*51, HLA-C*w01, and HLA-C*w07. The results indicated that HLA-B*18 binds to peptides with the highest affinity among the examined alleles, while HLA-B*51 binds to a greater number of peptides with the lowest affinity [62]. Self-peptides with high affinity to HLA will promote tolerance, while those with low affinity will not elicit an immune response. However, peptides with intermediate affinity to HLA may pose a problem since they are inefficient at developing tolerance while inducing an immunological response. Therefore, lower-affinity binding of peptides by HLA-B*5101 may cause predisposition to the development of distinct autoimmune responses [62]. 

Research aimed to investigate the low-affinity binding of HLA-B*51 and its potential involvement in BD showed that only HLA-B*5101, but not HLA-B*5201, is associated with BD. This suggests that the peptide presentation function may play a crucial role. The two variants differ in only two residues at positions 63 (Asp63Glu) and 67 (Phe67Ser), located in the B pocket of the peptide-binding groove, which separates the peptidomes of these two HLA alleles [63,64]. A comparative study of the molecular structure and peptide-binding dynamics of two HLA-B alleles, HLA-B*51 and HLA-B*52, using peptides (YAYDGKDYI, LPRSTVINI, and IPYQDLPHL) revealed that the HLA-B*51-bound peptides exhibited greater fluctuation and, consequently, looser binding. This resulted in less selective binding when compared to HLA-B*52 [64]. 

An analysis of the polymorphic amino acid positions of HLA-B revealed that positions 97, 116, 152, and 67 individually exerted an independent influence on the risk of developing BD [43]. Of particular interest are residues 97, 116, 152, and 67 within the HLA-B protein, all of which are situated within the MHC-I antigen-binding groove. These four MHC-I residues directly interact with six out of nine peptide residues. Residues 67 and 116 are especially critical as they serve as anchor residues, substantially influencing the peptide specificity of MHC-I antigen-binding grooves through their interactions with peptide positions P2 and P9, respectively [65]. Notably, positions 67 and 116 also play a pivotal role in determining the interactions between HLA-B molecules and KIR3DL1 and KIR3DS1, which are instrumental in regulating the activation of NK cells and CD8+ cytotoxic T lymphocytes [66]. Additionally, residue 67, as mentioned above, is one of two residues in which HLA-B*51 differs from the closely related HLA-B protein HLA-B52, which has no discernible impact on BD risk.

#### 3.3.3. Other HLA Class I Alleles

Previous research has identified significant associations between BD and specific HLA class I alleles other than HLA-B*51. Ombrello et al. [43] demonstrated that HLA-B15 and *HLA-B27* independently contribute to BD risk. Conditional analysis also revealed the protective effect of *HLA-B49* on BD. In addition, conditional analysis and stepwise forward logistic regression on the complete dataset and the HLA-B51-negative subset highlighted that HLA-A*03 exerts a significantly protective effect against BD, with the identified risk factors being *HLA-B15*, *HLA-A26*, and *HLA-B27*. Al-Okaily et al. [34] found increased frequencies of *HLA-A26* and *HLA-A31* and a reduced frequency of *HLA-B15* in BD patients from Saudi Arabia. A separate study in Japan revealed a weak association of *HLA-A2602* and *HLA-B3901* with BD within HLA-B51-negative patients [21]. Kang et al. [67] identified enhanced frequencies of *HLA-A02:07*, *A26:01*, and *A30:04* and a decreased frequency of *A33:03* in BD patients. A study on German and Turkish BD patients revealed a significant association of *HLA-Bw4-80I* and HLA-A*26 among the HLA-B*51-negative cohort [68]. HLA-A*26, HLA-B*08, and A*25 are implicated as risk alleles, whereas HLA-B*58 and HLA-A*33 are reported to be protective in a UK population [69].

While HLA-B*51 has been extensively studied in the context of BD pathogenesis, it is crucial to acknowledge that a significant proportion of BD patients do not carry this allele. Thus, the role of antigen presentation remains pertinent for patients with various HLA class I alleles. Notably, HLA-A*26 has emerged as a risk variant in multiple studies, encompassing patients of different ethnic backgrounds. A detailed analysis of the polymorphic amino acid residues in HLA-A revealed significant and independent associations between residues 161 and 97 of HLA-A and BD [43]. These specific residues are located within the MHC-I antigen-binding groove, mirroring the risk amino acid residues identified in HLA-B*51. 

#### 3.3.4. Association of KIR3D Receptor Polymorphisms

KIR haplotypes have been associated with HLA class-I-associated diseases such as psoriasis, birdshot chorioretinopathy, and AS. Killer inhibitory receptors (KIR) are highly diverse molecules situated on chromosome 19q13.4. NK cells interact with HLA class I molecules which serve as ligands for their *KIR*. The presence of activating *KIR* genotypes has been associated with protection against infectious diseases but susceptibility to autoimmune diseases. Conversely, inhibitory *KIR* genotypes are linked to protection against inflammatory diseases. The interaction between the *KIR* on the NK and HLA class I molecules exemplifies genetic epistasis, where the presence of both the receptor and ligand is necessary for functional activity, while the presence of one without the other has no influence on effector cell activity [70]. 

When it comes to the association of KIR3D receptors with BD, the existing data are somewhat conflicting. In an extensive study involving 1799 BD patients and 1710 healthy controls from Turkey, with HLA-B*51 data, no significant association was found between the activating *KIR3DS1* or inhibitory *KIR3DL1* alleles and the risk of BD [71]. Intriguingly, among the subset of cases with ocular disease, an increased presence of activating *KIR3DS1* alleles was observed, irrespective of HLA-B*51 status.

Conversely, an analysis at the allele level of *KIR3DL1/S1* revealed that the combination of low-expressing *KIR3DL1/S1* alleles with *KIR3DS1* significantly heightened the risk of developing BD, while high-expressing *KIR3DL1/S1* alleles in combination with null-expressing *KIR3DL1* reduced the risk of the disease [69].

### 3.4. Gene Loci Related to IL23/T17 Pathways

#### IL23R-IL12RB2 Locus

The *IL23R-IL12RB2* locus is integral to the IL-12/IL-23 receptor, a crucial component for promoting Th1 differentiation and mediating IL-12 signaling. Previous GWA studies involving BD patients identified two significant single nucleotide polymorphisms (SNPs) within this gene: rs924080 [13] and rs1495965 [72]. Furthermore, another study corroborating the role of IL-23R in BD revealed that missense variants of this gene, which lead to reduced responsiveness to IL-23, exert a protective effect against BD. Intriguingly, these variants also offer protection against several other disorders, including psoriasis, AS, IBD, and Crohn’s disease (CD) [9,13,72].

### 3.5. Gene Loci Related to Innate Immunity 

#### 3.5.1. Association of MEFV Gene

Mutations within the *MEFV* gene are accountable for causing the recessive autoinflammatory disease known as familial Mediterranean fever (FMF). FMF is prevalent among Mediterranean populations, indicating a potential selective advantage. Previous studies have suggested that FMF-associated genetic variants might be a susceptibility factor for BD [73,74].

Kirino et al. [75] examined the *MEFV* gene variants using deep sequencing from GWAS data for their involvement in innate immunity in BD. A detailed examination of these genetic variants in the *MEFV* gene revealed a single FMF mutation, known as *p.Met694Val (M694V)*, that had a significant association with BD in Turkish patients even in a heterozygous state (odds ratio: 2.65–2.73) but not among Japanese patients. M694V is recognized as a highly penetrant FMF variant and is linked to more severe inflammatory symptoms in FMF. Moreover, this same genetic variant has been identified as a risk factor for AS, IBD, and severe hidradenitis suppurativa in the Turkish population, indicating that *MEFV* mutations including M694V may cause predisposition to inflammatory diseases [76]. The disease-modifying effect of *MEFV* gene variants is further supported by a study revealing the presence of *MEFV* mutations in 70.6% of neuro-Behçet’s disease with more prominent white matter involvement [77]. 

Existing evidence suggests that the *MEFV* variants associated with FMF are gain-of-function mutations, resulting in an increased responsiveness to bacterial products. In mice carrying the M694V mutation, there is enhanced activation of caspase-1, leading to an overproduction of interleukin-1β upon lipopolysaccharide stimulation [78]. 

I. Association of *MICA* gene

The MHC class I polypeptide-related sequence A (*MICA*) gene, located on chromosome 6 between the tumor necrosis factor (TNF) and the MHC-B genes, has also been implicated in BD. SNPs have been discovered in many BD patients within the *MICA* gene. *MICA* interacts with CD314, also known as natural killer group 2 member D (NKG2D), thereby activating the NK receptor. This receptor is expressed on various immune cells, including NK cells, natural killer T (NKT) cells, CD8+ T cells, and innate lymphoid cells (ILCs). This interaction serves to activate the immune system, potentially leading to tissue damage. A comprehensive meta-analysis conducted by Zhang et al. [79] demonstrated an association between the *MICA-A6* allele and BD. This allele can be considered a risk factor for the disease. Interestingly, the results also suggested that *MICA-A9, A5, A5.1*, and *A4* alleles exert protective effects against the development of BD [50,79].

#### 3.5.2. Association of FUT2 Gene

The fucosyltransferase 2 *(FUT2)* gene, which is involved in intestinal mucosal immunity has been associated with BD [80,81]. The association of the *FUT2* gene with BD was unveiled in the GWA studies conducted by Xavier et al. [80]. Their findings gained additional support from a meta-analysis that consolidated their data with a Turkish GWAS dataset focusing on Iranian patients. Furthermore, Xavier et al. [80] demonstrated that the *FUT2* gene serves as an independent genetic risk factor for BD, distinct from the HLA-B gene, and they found no evidence of epistasis.

The protein encoded by the *FUT2* gene, known as FUT2, plays a crucial role in the synthesis and secretion of the H antigen—a precursor of major ABO blood antigens—within the intestinal mucosa and bodily fluids. Owing to the genetic variability among humans, approximately 80% of individuals exhibit a secretor phenotype, denoting the presence of at least one functional *FUT2* allele. Conversely, the remaining 20% showcase a non-secretor phenotype [82]. The non-secretor phenotype, which has been linked to BD in the aforementioned study, is also associated with type 1 diabetes, CD, and rheumatic fever. Notably, this phenotype is correlated with a decreased susceptibility to infections caused by microorganisms like Helicobacter pylori, Campylobacter jejuni, and the Norwalk virus [80]. The Human Microbiome Project elucidated that the *FUT2* phenotype influences the abundance and diversity of Bifidobacterium in the microbiome. Individuals with a non-secretor phenotype exhibit a decrease in Bifidobacterium longum within their microbiome [83]. While further investigation is warranted to fully understand the impact of the *FUT2* gene on the gastrointestinal microbial flora, certain studies suggest that *FUT2* stands as one of the genes bridging environmental and genetic factors [80,81]. 

#### 3.5.3. Toll-Like Receptor Genes

The group of *TLR* genes offers further insights into the role of immune responses against bacteria in BD [81]. Among these genes, *TLR4* stands out as the most associated with BD within this family. SNPs within the *TLR4* gene have been linked to an elevated risk of various inflammatory disorders, including rheumatoid arthritis (RA), ulcerative colitis, and CD [84]. The connection between the TLR4 gene and BD has been established in Japanese patients [85]. Additionally, a study involving Korean patients revealed that the TAGCGGTAA haplotype exhibited a notably higher prevalence among HLA-B*51+ BD patients in comparison to healthy controls [84]. Kirino et al. [75] reported that two *TLR4* variations, namely *p.Asp299Gly (D299G)* and *p.Thr399Ile (T399I*), conferred a protective effect against BD. Interestingly, these hyporesponsive variants had previously been associated with an increased risk of CD.

Furthermore, investigations involving the Han Chinese population revealed a linkage between the *TLR2* gene and BD. Specifically, two *TLR2* genotypes—*rs2289318* and *rs3804099*—were found to be more prevalent in ocular BD patients compared to healthy individuals [86].

#### 3.5.4. TNFAIP3

The tumor necrosis factor alpha-inducible protein 3 (*TNFAIP3*) gene has recently been debated as a BD-associated gene or related to a familial BD-like autoinflammatory syndrome of haploinsufficiency of A20 (HA20) [87,88,89]. The *TNFAIP3* gene encodes the A20 enzyme, which modulates inflammatory reactions via TNF, TLR, or NOD2-induced nuclear factor kappa-light-chain-enhancer of activated B cells (NF-κB) signaling [90,91]. Polymorphisms in *TNFAIP3* are associated with autoimmune diseases (systemic lupus erythematosus (SLE) [92], RA [93], multiple sclerosis (MS) [94], and psoriasis [95]). Han Chinese BD patients with SNPs (e.g., *rs9494885, rs10499194, rs7753873*) exhibit elevated BD risk [88]. Monogenic variants like nonfunctional TNFAIP3 forms lead to early-onset BD-like syndrome (HA20) due to A20 haploinsufficiency [87]. HA20 results from loss-of-function mutations prompting increased NF-κB signaling, intensifying immune cell-driven inflammation [96]. HA20 has oral, genital ulcers, GI involvement and arthritis similar to BD. However, HA20, is more frequent in women (1:2 male-female ratio), emerges earlier in childhood (median age 5.5). Typically attacks are accompanied by recurrent high fever. Although the prevalence is low, HA is globally evenly distributed in contrast to BD. and the presence of HLA-B*51 antigen is low [97].

#### 3.5.5. STAT4

Signal transducer and activator of transcription-4 (*STAT4*) is associated with an elevated risk of various autoimmune diseases, including primary Sjögren’s syndrome, RA, and SLE [12,98]. *STAT4* was shown to be associated with BD in a GWA investigation of Turkish individuals [45]. One of the related SNPs revealed in GWA research on Han Chinese patients, rs897200 risk allele A, was linked to more severe BD symptoms, including increased STAT4 expression, greater interleukin 17 (IL-17) production, and more severe clinical signs [99]. 

#### 3.5.6. Other Associated Genes

Some more gene loci associated with susceptibility to this disease were discovered in GWA studies of BD patients. These genes include, but are not limited to, the *IL-10* gene, which is a cytokine that is reduced in the blood of BD patients. In studies from Turkey and Iran, one SNP in the *IL-10* gene, *rs1518111*, was linked to BD, and the risk allele A (found in Turkish GWA studies) was found to result in a 35% lower expression of IL-10 in monocytes [15,33,34,51]. Additionally, the CD40 gene featured two linked SNPs, *rs4810485* and *rs1883832*. These SNPs had a predisposing effect in TT genotypes and a protective effect in GT genotypes for rs4810485 [34,52]. BD susceptibility involves various genomic regions, influenced by both genetic and environmental factors. Understanding their interplay is crucial for future therapies and accurate diagnostics [34].

### 3.6. Antigens in Behçet’s Disease

The HLA class I associated disease hypothesis suggests that increased antigenicity of an HLA-B*51-presented peptide can trigger CD8+ T cell recognition of a self-protein, potentially initiating the disease. This concept aligns with findings of increased CD8+ T cells in the aqueous humor and the presence of oligoclonal and clonal expansions of CD8+ T cells in the peripheral blood of BD patients, providing supportive evidence [100,101]. In one study, 67% of clinically active BD patients displayed oligoclonal expansions in both CD4+ and CD8+ T cells, with five out of nine patients exhibiting a CD8+ T cell clone featuring the Vb5.1 chain [101]. Another analysis of the peripheral blood mononuclear cells (PBMCs) found one or more CD4+ or CD8+ T cell clones in 57% of patients. Longitudinal research revealed that all patients with active disease harbored a specific T cell clone associated with the disease [100]. 

Self-peptides produced from an intracytoplasmic protein that have molecular similarity to a peptide previously identified as a structure of a harmful bacterium may elicit an immune response in CD8+ T cells, although the specific self-peptides responsible for BD development remain unidentified. Research has predominantly focused on the environmental antigens implicated in BD autoimmunity, including bacterial peptides (e.g., *Streptococcus sanguinis*, *Mycobacterium tuberculosis*, *Helicobacter pylori*) and viral antigens (e.g., HSV1, cytomegalovirus, hepatitis, Epstein-Barr virus) [102,103]. Notably, the 50% similarity between heat shock protein-65 (HSP-65) in *Streptococcus sanguinis* and *Mycobacterium tuberculosis* and the human 60-kDa heat shock protein (HSP-60) suggests a potential BD trigger [102,104,105]. The cross-reactivity between human and bacterial HSP may induce autoreactive T cell proliferation or autoimmunity [105,106]. BD patients exhibit antibodies against HSP-65, which cross-react with oral epithelium antigens. *Mycobacterium* HSP-65 epitopes (amino acids 111-25, 154-172, 219-233, and 311-326) trigger B and T cell reactions [104,107,108,109,110]. When BD patient T cells were activated using a peptide pool derived from the 65-kDa HSP, four significant immunotopes (111-125, 154-172, 311-325, and 219-233) were identified [111]. These *M. tuberculosis* HSP immunotopes stimulated δT cell activation and proliferation in 76% of the patients, while αβ CD8+ T cells were not elevated [112].

Recent Turkish research found autoantibodies against neurofilament-M, an intracellular protein, in BD patient sera. The peptide structure of this protein exhibited homology with three immunotopes (amino acids 111-126, 213-232, and 304-363) of *M. tuberculosis* HSP and (161-176, 304-363, and 340-359) of *S. sanguinis* HSP. Additionally, BD patient blood shows immunological reactivity to the self-antigen neurofilament medium, sharing amino acid similarity (111-126, 213-232, 304-363) with *Mycobacterium* HSP-65 [104,107,108,109,110]. Sera containing neurofilament-M autoantibodies responded to bacterial HSP [109]. Other studies have demonstrated autoreactive CD8+ T cells in BD upon stimulation with MICA peptide and *S. sanguinis* HSP [105,110,113].

Studies indicate that BD patients often exhibit disrupted oral flora and poor oral health, with the plaque index being correlated with disease severity. Antibiotics can sometimes help control disease recurrence [3,107,114]. Among the investigated bacteria, *S. sanguinis* yielded the following findings: (1) BD patients have a higher proportion of these bacteria in their oral flora compared to healthy individuals. (2) BD patients exhibit hypersensitivity to antigens from this bacterium. (3) Gnotobiotic animal models inoculated with these bacteria displayed BD-like symptoms. (4) Gamma-delta (γδ) T cells from BD patients react to antigens from S. sanguinis.

In the context of BD, one virus that has been extensively researched is HSV1. It is noteworthy that HSV1 DNA has been identified not only in BD lesions located in the oral and vaginal regions but also in blood samples obtained from BD patients. Moreover, BD patients exhibit elevated levels of antibodies against HSV1 in comparison to healthy individuals. Interestingly, when animal models were inoculated with this virus, it resulted in the manifestation of BD-like symptoms [60,107,115].

### 3.7. Exploring the Involvement of Diverse Immune Cells in Behçet’s Disease

**Figure 3:**
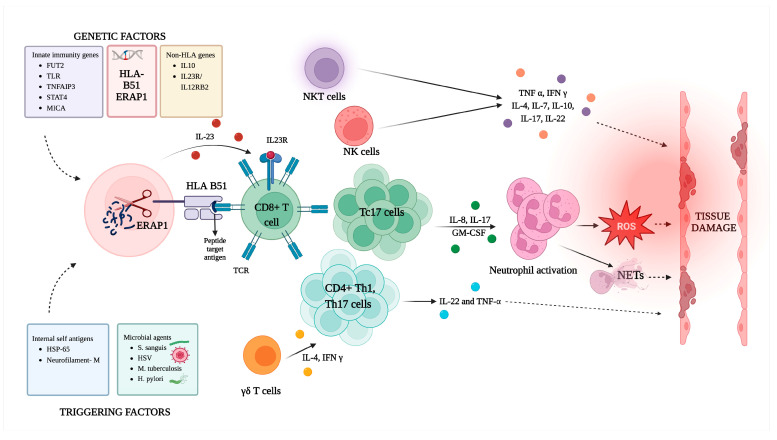
Schematic illustration of pathogenesis in Behçet’s disease: This schematic provides an illustration of the genetic determinants and potential triggering antigens that activate an immune cascade when self-peptides, mimicking environmental antigens, are presented to CD8 T cells. This activation is further triggered by the release of IL-23. Immune dysregulation leads to the release of cytokines by various immune cells such as Tc17, Th17, NKT, and NK cells. The dominant cytokines involved in this process, including IL-23, IL-17, TNF-alpha, IFN-gamma, and IL-8, play a pivotal role in activating neutrophils, generating reactive oxygen species, and triggering the formation of neutrophilic extracellular traps. Subsequently, this immune response can lead to tissue damage. (Created using BioRender.com, accessed on 14 May 2023).

The innate and adaptive immune systems both have a role in the pathogenesis of BD. The activation of adaptive immunity in BD may be coupled with the activation of innate immunity by pathogen-associated molecular patterns (PAMPs) from tissues exposed to the external environment, or damage-associated molecular patterns (DAMPs) generated by tissues under stress (Figure 3). The ratios of Th1/Th2 and Th17/Th1 and cytotoxic CD8+ T cells are raised in the adaptive immune response, while the number of regulatory T cells (Tregs) is decreased [11,116]. Other cell types implicated in the pathophysiology of BD include neutrophils, γδ T cells, and NK cells [117] This section will discuss the various immune cells involved in the pathogenesis of BD.

#### 3.7.1. Cytotoxic T Cells (CD8+ T Cells)

HLA-B*51 is the key genetic factor in BD and plays a crucial role in presenting peptides to CD8+ T cells. Consequently, CD8+ T cells are implicated in BD’s pathogenesis. This is supported by increased CD8+ T cells in the aqueous humor and the presence of oligoclonal and clonal expansions of CD8+ T cells in the peripheral blood of BD patients [56,57]. CD8+ T cells release various cytokines, including IL-17, IL-8, and granulocyte–macrophage colony-stimulating factor (GM-CSF), which have diverse effects, such as enhancing neutrophil activation and facilitating the interaction between innate and adaptive immunity in BD’s immunopathogenesis [50]. In active HLA-B*51+ BD patients, blood samples exhibit a higher proportion of CD8+ T cells compared to healthy individuals and inactive BD patients [118]. The number of these cells is increased in the aqueous humor of BD patients with ocular involvement [119]. Studies also indicate that in BD skin lesions, IL-17-secreting T cells predominantly originate from CD8+ T cells rather than CD4+ cells [116]. 

#### 3.7.2. T Helper 1 Cells

T helper 1 (Th1) cells play a significant role in BD. Comparing active BD patients to both inactive BD patients and healthy individuals reveals an increase in Th1 cells and their associated cytokines, such as interferon gamma (IFN-γ). In ocular non-infectious inflammations like BD, Vogt–Koyanagi–Harada (VKH), and sarcoidosis, there is a notable elevation in Th1 cell infiltration [120]. Moreover, research by Ye et al. [121] highlighted the impact of reduced B and T lymphocyte attenuator (BTLA) in the disease’s pathogenesis. This reduction amplifies the activity of Th1 and Th17 cells, worsening ocular inflammation in BD. Exploring this pathway could offer promising avenues for new BD uveitis treatments. Notably, VKH syndrome, the second major cause of uveitis, is unaffected by the decrease in BTLA. Additionally, the mRNA expression of Th1-related cytokines is notably higher in BD patients’ mucocutaneous lesions [1,120,121,122].

#### 3.7.3. T Helper 17 Cells

Th17 cells have garnered significant attention for their involvement in various autoimmune and inflammatory disorders like psoriasis, MS, RA, and IBD [80]. In a mouse model, the differentiation of Th17 cells from naive T cells is stimulated by IL-6 and transforming growth factor-β (TGF-β). Additionally, TNF-α and IL-1 enhance Th17 differentiation when these cytokines are present. What is particularly intriguing is that while TGF-β typically inhibits most T cell responses and promotes the generation of Tregs, it paradoxically increases the population of Th17 cells [81]. In a study conducted by Xu et al. [123], it was revealed that Treg cells can transform into proinflammatory Th17 cells when exposed to IL-6 [82]. Consequently, Tregs lose their ability to suppress Th17-induced inflammation and may even exacerbate it. This complex interplay underscores the significance of Th17 cells in immune-related diseases.

Diverse studies have presented varying perspectives concerning the role of TGF-β in the differentiation of Th17 cells. Some research contends that TGF-β is imperative for the differentiation of Th17 cells, particularly when employing naïve T cells derived from cord blood. Furthermore, the concentration of TGF-β emerges as a critical determinant in its functionality, where diminished levels facilitate Th17 differentiation, and elevated concentrations promote differentiation of Tregs. Conversely, conflicting research proposes that TGF-β, despite being a requisite for Th17 differentiation in murine models, exerts an inhibitory effect on the genesis of Th17 cells in human subjects. This disparity poses a challenge in the translational applicability of findings from murine disease models to human cases. In conclusion, it is imperative to conduct further rigorous investigations to comprehensively elucidate the nuanced influence of TGF-β on Th17 cell differentiation and its ramifications across a spectrum of pathological conditions [123,124,125,126,127,128].

Initially, Th1 cells were presumed to be the central players in BD. However, a substantial body of research has demonstrated an elevated presence of Th17 cells in BD patients, signifying their role in BD pathogenesis [129,130]. BD patients exhibit increased counts of both Th1 and Th17 cells. Moreover, in BD patients, there is a notable upregulation of Th17-associated cytokines, including IL-22 and TNF-α. Furthermore, the transcription factor essential for Th17 cell differentiation, retinoic acid-related orphan receptor γ (RORγt), is found at increased levels in BD patients [122,129,131]. In summation, Th17 cells constitute a crucial component in the immunopathological framework of BD, offering valuable insights into the disease’s etiology and holding potential for the identification of innovative therapeutic strategies.

#### 3.7.4. Natural Killer T Cells

Upon activation, NKT cells exhibit the secretion of cytokines, including TNF, IFN-γ, IL-4, IL-10, and IL-17. The specific cytokine profile produced is contingent upon factors such as the NKT cell subtype, signal intensity, and the antigen-presenting cells (APCs) involved. NKT cells and their deficiencies serve as valuable diagnostic markers for various diseases and represent an area of active exploration for potential therapeutic and preventive strategies [132,133,134,135].

In BD patients with ocular involvement, there is an increase in the number of NKT cells, notably the CD8+CD56+NKT subset in the aqueus humor [119]. However, the data regarding changes in the number of NKT cells in the peripheral blood of BD patients present conflicting results, with some studies indicating an increase while others report a decrease in their numbers [136]. Furthermore, NKT cells exhibiting high IFN-γ expression are elevated in the cerebrospinal fluid (CSF) of BD patients experiencing neurological symptoms during the active phase of the disease, with a subsequent reduction in these cells when patients transition into remission. Notably, NKT cell activity in the blood displays a concurrent decrease [137].

#### 3.7.5. Gamma-Delta (γδ) T Cells

Gamma-delta (γδ) T cells, distinct from conventional αβ T cells, are integral to the immune response. Typically, γδ T cells lack both CD4 and CD8 markers and do not rely on MHC class I or II molecules for antigen recognition [138]. During infections, these cells significantly increase in the bloodstream. In mouse models, γδ T cells have emerged as the primary source of IL-17, a potent pro-inflammatory cytokine critical in conditions such as BD, particularly during the onset of inflammation.

Two distinct subsets of γδ T cells secrete IL-17. Firstly, natural γδ 17 T cells, known as natural Tγδ17 cells, circulate in the bloodstream, becoming effector cells upon encountering infections. They secrete various cytokines, including IL-17, as part of their immune response. Secondly, induced γδ T17 cells rapidly propagate in response to infection, operating without the need for clonal expansion, yet they significantly contribute to the immune response against pathogens. This dual secretion mechanism of IL-17 underscores the versatility of γδ T cells in immune regulation.

Moreover, γδ T cells exhibit a unique ability to function as professional APCs. Multiple investigations have demonstrated their proficiency in activating other immune cells, including both CD4+ and CD8+ T cells, within lymph nodes [139,140,141,142].

γδ T cell fractions exhibit noteworthy differences in active BD patients when compared to both healthy individuals and those with inactive BD [75]. These unique T cells are characterized by their release of substantial quantities of cytokines, which result in neutrophil hyperactivity and the induction of Th1 and Th17 cells [118]. These cells release significant quantities of cytokines, causing neutrophil hyperactivity and the induction of Th1 and Th17 cells [143]. In the context of BD, it is of particular interest that the Vγ9Vδ2 T cell phenotype experiences a significant elevation in patients. These cells not only produce various inflammatory cytokines, including IL-17, but also cytotoxic molecules such as granzyme A, which are known to play a pivotal role in the pathogenesis of the disease [144,145].

The association between γδ T cells and BD was initially established in the early 1990s, when researchers observed higher levels of γδ T cells within the PBMCs of a group of BD patients [146,147]. When stimulated, these cells are known to release IFN-γ and TNFα [148]. Interestingly, γδ T cells are believed to have a significant impact on the adaptive immune response by secreting cytokines like interleukin-4 (IL-4) or IFN γ, potentially influencing the immune response toward the Th2, Th17, and Th1 CD4+ T cell phenotypes. Additionally, γδ T cells have been shown to establish efficient interactions with neutrophils and monocytes during acute microbial infections in response to bacterial antigens. Their natural functional diversity and adaptability render them significant contributors to disorders like BD, which affect various compartments of the body.

However, despite the potential significance of γδ T cells in BD, the evidence regarding their role in the disease remains somewhat contradictory. While some studies have reported an increase in the population of γδ T cells in BD patients [146,147,148,149], others have not found a substantial elevation of this specific cell category in PBMCs [140,150,151].

#### 3.7.6. Regulatory T (Treg) Cells

Regulatory T cells constitute a specialized subset of T cells characterized by the expression of the forkhead box protein P3 (FoxP3), and they play a critical role in maintaining immunological balance while preventing autoimmune responses [152,153,154,155]. It is evident that any malfunction or reduced quantity of these Tregs can lead to the development of autoimmune disorders, as exemplified by conditions like MS, RA, SLE, and myasthenia gravis [152]. 

The role of Tregs in BD has been the subject of investigation, yielding somewhat conflicting results. Some studies suggest a decrease in the number of Treg cells in BD patients, and this reduction is correlated with disease activity, potentially attributed to increased levels of the cytokine IL-21 [156]. Conversely, another study focusing on the CSF of BD patients with neurologic involvement reported an increase in Treg cell numbers compared to individuals with noninflammatory neurological conditions. However, this increase was associated with a deficiency in suppressing T helper responses, possibly indicating a decline in Treg cell functionality. Furthermore, under inflammatory conditions marked by the presence of IL-1β and IL-2, these Treg cells were proposed to undergo transformation into Th17 cells [157]. The dual nature of Treg cells in BD, where their numbers may increase in specific contexts, but their functional effectiveness may diminish, highlights the intricate immunological dynamics at play in this disorder. 

#### 3.7.7. Natural Killer Cells

Natural killer cells (NK cells) constitute a vital component of the innate immune system. NK cells can interact with HLA class I molecules, which are < z < z < Zz < ligands for their KIR receptors. In several autoimmune disorders such as SLE, Sjögren’s syndrome, MS, and RA, there is a consistent pattern of both diminished numbers and impaired functionality of NK cells.

NK cells are categorized into two types within the body:

1. CD56brightCD16 NK cells, primarily found in the lymph nodes, specialize in cytokine release.
2. CD56dimCD16+ NK cells, predominantly present in the blood and inflamed sites, primarily exert cytotoxic effects using proteins like perforin and granzyme, with a diminished capacity for cytokine release compared to the former category [158,159].

Similarly, in BD, a reduction in the quantity of NK cells in the bloodstream is observed, possibly attributed to their migration to inflamed sites. Notably, among the NK cell population, there is an increase in CD56bright NK cells, which are known for cytokine production, particularly IFN-γ [160,161].

NK cells can also be categorized based on the cytokines they produce, falling into five distinct groups: NK-1, NK-2, NK-17, NK-reg, and NK-22, each associated with the release of specific cytokine types such as Th1, Th2, IL-17, IL-10, and IL-22, respectively. Research findings in BD patients reveal an elevated proportion of NK-1 cells, while the numbers of NK-2, NK-17, and NK-reg cells are diminished. Furthermore, investigations have illuminated a notable shift in the NK-1/NK-2 ratio between the active and remission phases of BD. During the active phase, NK-1 predominates, promoting a Th1 immune response, whereas in the remission phase, NK-2 becomes dominant, tilting the immune response toward Th2 activation [117,162,163].

#### 3.7.8. Neutrophils

BD patients frequently exhibit heightened neutrophil activity and an increased neutrophil count, a phenomenon supported by various investigations. Additionally, granulocyte colony-stimulating factor (G-CSF) levels are elevated in active BD patients, contributing to increased neutrophil apoptosis [164]. Moreover, several studies have reported an elevated neutrophil-to-lymphocyte ratio in BD patients, with Djaballah Ider’s research highlighting its potential as an indicator of BD and disease severity [165,166]. Le Joncour et al. [167] have demonstrated elevated levels of neutrophil extracellular traps (NETs) and associated markers like myeloperoxidase (MPO) and cell-free DNA (CfDNA) in BD patients, implicating their role in the disease’s pathophysiology. Notably, NETs have been observed in BD papulopustular lesions [116]. Neutrophils accumulate significantly in all types of BD lesions, including those affecting the skin, mucocutaneous regions, and eyes. In BD patients, these hyperactive neutrophils induce tissue damage via the oxidative stress mediated by reactive oxygen species (ROS) [168].

## 4. Conclusions

In conclusion, BD exhibits an intricate pathogenesis, driven by the interplay of autoinflammatory and autoimmune components. Our review highlights the pivotal role of genetics, particularly the HLA class I allele HLA-B*51. However, the genetic landscape of BD is diverse, as a spectrum of non-HLA genes, including *ERAP1*, *IL-10*, *IL23R/IL-12RB2*, and various innate immunity genes, have also been implicated in disease susceptibility.

GWA studies have revealed the prominence of *ERAP1* alongside HLA-I. The epistatic link between HLA-B*51 and *ERAP1* highlights how specific *ERAP1* polymorphisms become risk factors only in the presence of the HLA-B*51 allele. These genetic variations can modulate antigen presentation and enzymatic activity, thereby influencing the HLA-I peptidome and binding affinity. This interaction may shape the immune response, potentially triggering distinct autoimmune responses when peptides of intermediate affinity to HLA are involved.

It is noteworthy that the presence of HLA-B*51 and its combination with the ERAP1-Hap10 variant is notably absent in the majority of BD patients. Further research is imperative to elucidate the underlying mechanisms at play in the remaining subset of BD patients.

BD pathogenesis also involves a dynamic interplay between innate and adaptive immunity. The activation of adaptive immunity, characterized by imbalances in the Th1/Th2 and Th17/Th1 ratios, coupled with increased cytotoxic CD8+ T cells and decreased Treg cells, underscores the complexity of the immune responses. Cytotoxic T cells, particularly CD8+ T cells, have garnered attention as they release an array of cytokines, including IL-17, IL-8, and GM-CSF, leading to neutrophil activation and facilitating the collaboration of innate and adaptive immunity. Other cells such as neutrophils, γδ T cells, and NK cells, also play a role.

This comprehensive analysis provides valuable insights into the role of genetics and immunity underlying BD pathogenesis. However, it is essential to recognize that the precise roles of specific immune cell types, such as cytotoxic CD8+ T cells, neutrophils, γδ T cells, and NK cells, and their interactions in BD pathogenesis warrant more in-depth investigation. Given these limitations, our review highlights the need for continued research into BD’s pathogenesis. Further studies incorporating advanced genomic techniques and comprehensive immune profiling will be crucial to unravel the complexities of this disease. Such investigations not only have the potential to refine our understanding of BD but also pave the way for more targeted and effective diagnostic and therapeutic strategies, ultimately improving the management and treatment of this disorder.

## Data Availability

No new data were created or analyzed in this study. Data sharing is not applicable to this article.
